# Recent development in functional nanomaterials for sustainable and smart agricultural chemical technologies

**DOI:** 10.1186/s40580-022-00302-0

**Published:** 2022-03-02

**Authors:** Chen Shao, Huawei Zhao, Ping Wang

**Affiliations:** 1 Bio-Nanotechnology Research Institute, Ludong University, Yantai, 264025 Shandong China; 2 School of Food Engineering, Ludong University, Yantai, 264025 Shandong China; 3 Department of Bioproducts and Biosystems Engineering, University of Minnesota, St Paul, MN 55108 USA

**Keywords:** Nanotechnology, Nanomaterial, Agrochemical, Nanopesticide, Nano-fabricated fertilizer, Nano activity-based growth promoter

## Abstract

New advances in nanotechnology are driving a wave of technology revolution impacting a broad range of areas in agricultural production. The current work reviews nanopesticides, nano-fabricated fertilizers, and nano activity-based growth promoters reported in the last several years, focusing on mechanisms revealed for preparation and functioning. It appears to us that with many fundamental concepts have been demonstrated over last two decades, new advances in this area continue to expand mainly in three directions, i.e., efficiency improvement, material sustainability and environment-specific stimulation functionalities. It is also evident that environmental and health concerns associated with nano agrochemicals are the primary motivation and focus for most recent work. Challenges and perspectives for future development of nano agrochemicals are also discussed.

## Introduction

The rapidly growing demands in sustainable economy set forth grand challenges to current agricultural industry that have traditionally relied heavily on agrochemicals, including synthetic pesticides and fertilizers. Up to now, the global use of pesticides and fertilizers have reached over 4.1 and 125 million tons, respectively [1, 2]; however, the traditional way of administering agrochemicals is generally inefficient. For pesticides, which are generally synthetic organic compounds with high hydrophobicity and often poor chemical stability, it is estimated that only less than 1% of the applied dosage served the purpose, while the majority get lost to environment via volatilization, degradation, and photolysis in traditional application scenarios [3, 4]. A similar efficiency is also estimated for conventional fertilizers, which provide essential macronutrients (N, P, K, Mg, Ca, S, Si) and micronutrients (Fe, Cu, Zn, Mn, B, Mo, Ni, Na, Cl) for plant growth [5]. For example, volatilization and leaching could take away over 50% of nitrogen (N) content in traditional fertilizers [6]. Such a low efficiency not only increased the cost of production, but also generated widely concerned environmental and health impacts [7]. Reducing the amount and improving the use efficiency of agrochemicals by applying smart and precision agricultural technologies have therefore attracted extensive research interests [8], and release behavior control, chemical stability retention, and targeting capability are the fundamental pursuits, where nanotechnology is playing a crucial role [7, 8].

**Fig. 1:**
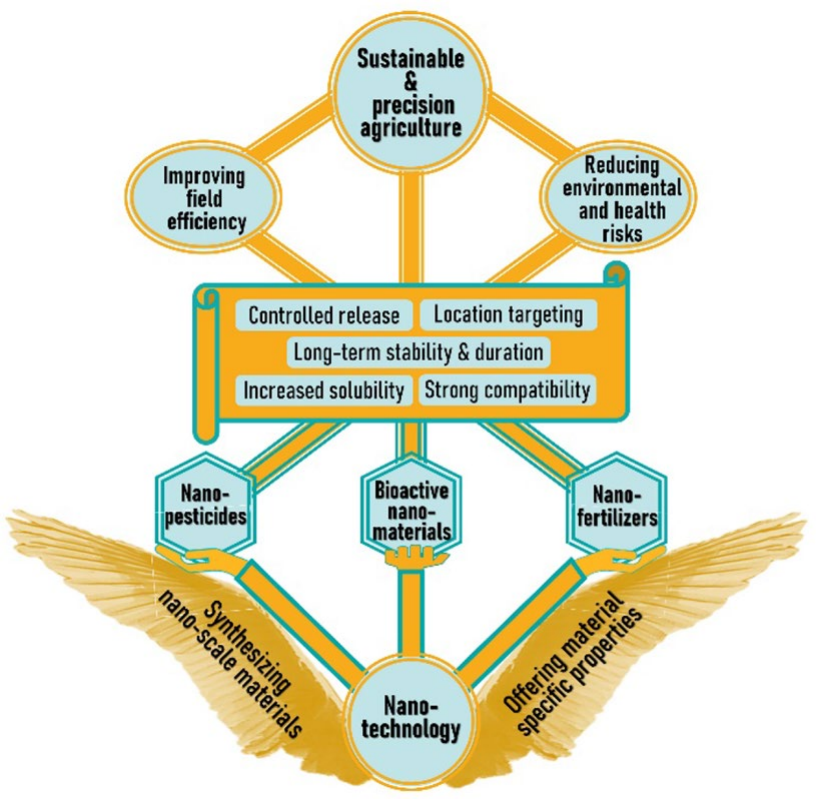
Topics and Concepts Entailed in Nanoscale Agrochemicals for Sustainable and Precision Agriculture Technologies

Nanotechnology focuses on synthesis, manipulation and functionalization of nanomaterials [9]. Nanotechnology has made great impacts in recent advances in all the major technological areas including biomedical engineering, food science, IT, etc. [10–19]. For precision agriculture, agrochemicals prepared in different formats of nanomaterials promise a variety of appealing characteristics, including controlled and stimulation-regulated release rate, location- or time-specific targeting, long-term stability and duration, increased solubility, enhanced compatibility, etc. [7, 8]. Both structural design and material composition of the nanomaterials can be manipulated for optimal in-field functionality and activity, affording the technology sustainability and precision characteristics (Fig. 1). Agricultural nanotechnologies have been proposed and substantially examined over the last two decades, with tens of reviews published from various perspectives. This review will focus mostly on research published in the last five years, examining new advances in synthetic strategies, functionalities, and applications of nanoscale agrochemical technologies. It appears that nano-fabricated pesticides and fertilizers for improved field efficacy continued to be the focus in recent years. Attention is particularly evident on topics such as material selection, preparation optimization, structural design, and stability enhancement, toward sustainability and environmental considerations. These will be discussed in the first two parts of the review. At the same time, a growing interest has also been found in developing biologically active nanomaterials, instead of nanocarriers of other active or nutritional agents, to promote crop growth, seed germination, insect resistance, microenvironmental regulation, etc. This aspect will be discussed in the third part. Analytical nanotechnologies such as those targeting pathogen and pesticide/herbicide residue detections constitute another major category of nanotechnologies for agricultural production, and have been reviewed previously [20–27], will not be covered in this work.

## Nanopesticides

Conventional pesticides are generally synthetic organic compounds with high hydrophobicity. Traditional processing and formulation often require organic solvents which cause environmental pollution and health risks [14]. Nanomaterials can replace organic solvents in their processing and formulations, bringing in other beneficial properties at the same time such as improved adhesion to crops, stability against degradation and controlled release for improved efficiency. Controlled release is probably the most attractive trait of nano-fabricated pesticides (nanopesticides). We may classify nanopesticides into two categories according to the patterns of release rate regulation, i.e. sustained-release and stimulated-release. While the former focuses on slowing down the release rate and thus extending the lifetime of pesticides, the latter is directed toward more vigorous regulation of the pesticide release by responding to environment factors such as light, temperature, pH, etc.

### Sustained-release nanopesticides

Sustained-release of pesticides from nanocarriers mainly occurs in form of passive diffusion, capsule erosion, or osmotic-driven permeation. In the fabrication of sustained-release nanopesticides, major challenges are how to achieve high loading efficiency and optimal release rate of the active ingredients. That is closely related to material selection, processing strategy, and final structural layout. Entrapment and adsorption are two basic approaches in formulation of sustained-release nanopesticides. Table 1 summarizes major types of sustained-release nanopesticides reported recently.

**Table 1:** Typical materials and preparation strategy applied for sustained-release nanopesticides

Matrix material/ active ingredient	Fabrication strategy/method	Refs.
Sodium Alginate /Imidacloprid	Entrapment/Emulsion cross linking technology	[31]
Starch Acetate /Avermectin	Entrapment/Emulsion-solvent evaporation (PME technology)	[32]
Hypromellose Acetate Succinate/Abamectin	Entrapment/Nanoprecipitation	[33]
Sodium Lignosulfonate and CTAB /Avermectin-	Entrapment/Electrostatic Self-Assembly	[36]
Benzoyl Lignin /λ-Cyhalothrin	Entrapment/Nanoprecipitation	[37]
PLA /Chlorantraniliprole	Entrapment/Emulsion-Solvent Evaporation (PME technology)	[40]
PLGA /Pyraclostrobin	Entrapment/Emulsion-Solvent Evaporation (microfluidic technology)	[41]
Synthetic Polymer/Difenoconazole, Prochloraz, Pyraclostrobin, and Tebuconazole	Entrapment/ “Hat”-Shaped Janus Carriers Formed by Emulsion Interfacial Polymerization	[42]
Synthetic Castor Oil-Based Polyurethanes /Avermectin	Entrapment/Emulsion-Solvent Evaporation	[43]
Calcium Carbonate /Validamycin	Entrapment/Reversed-phase Microemulsion	[47]
Active Carbon/2,4-Dichlorophenoxyacetic Acid Sodium	Physical Adsorption	[48]
Porous Silica Nanosphere /Imidacloprid		[49]
Zirconium-based MOF /Pyrethroids		[59]
Iron-Based MOFs /Chlorantraniliprole		[60]
Aluminum-Based MOFs/ Azoxystrobin and Diniconazole		[61]
Iron-based MOFs /Diniconazole		[62]
Fe_3_O_4_-MOF Core–shell Nanocarrier/Imidacloprid		[63]
Zinc MOF /*ortho*-Disulfides	Entrapment With Further Modification with β-Cyclodextrin	[64]

#### Nanopesticides prepared by entrapment

**Fig. 2:**
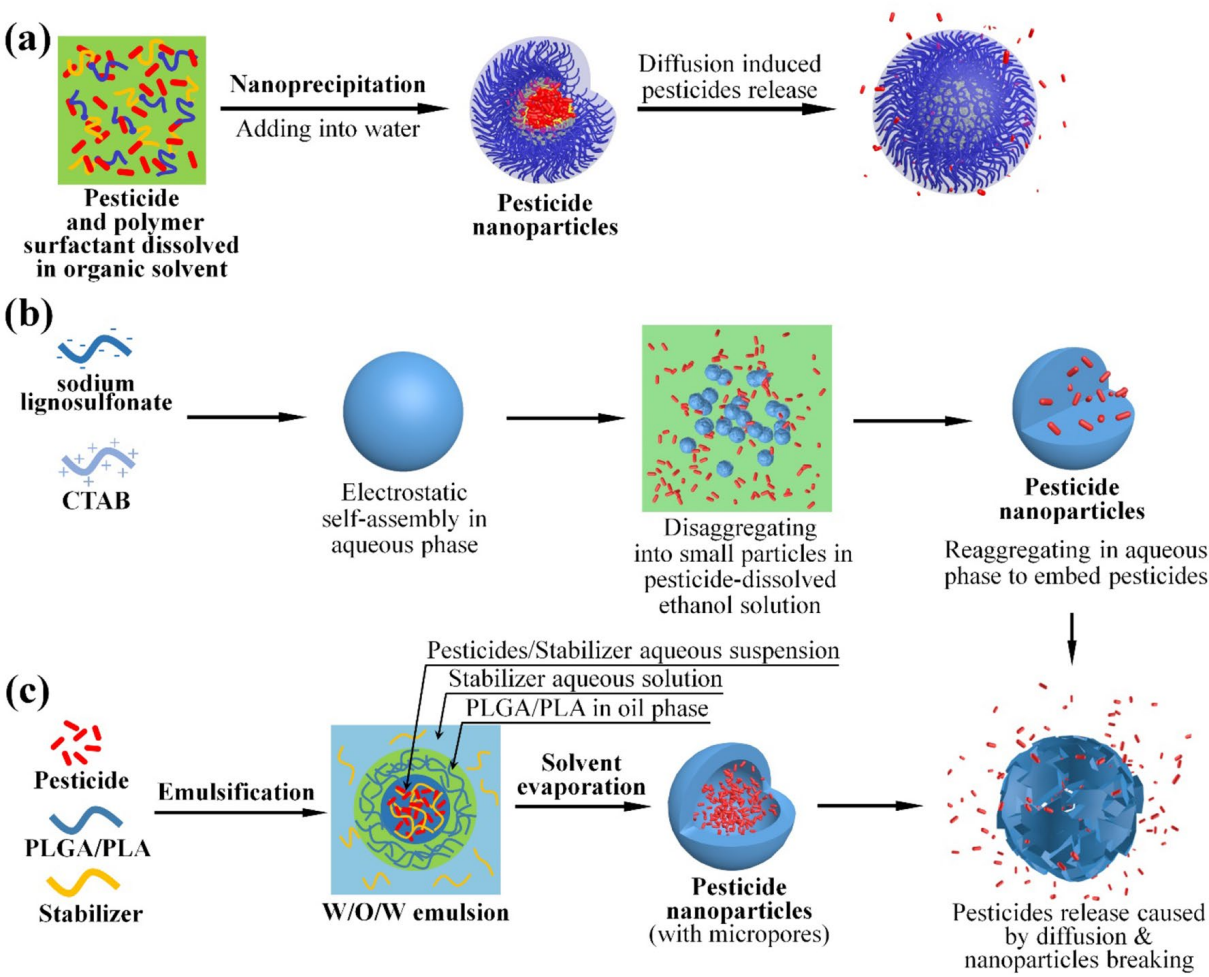
Different Synthetic Strategies and Action Mechanisms of Polymer-Based Sustained-Release Nanopesticides. **a** Nanoprecipitation [33, 37]; **b** Electrostatic self-assembling process [36]; **c** PLGA/PLA nanopesticides prepared by emulsion-solvent evaporation method [40, 41]

Entrapment is a popular and efficient strategy for preparation of nanopesticides. Polymeric matrices provide an ideal network structure for pesticide entrapment [28] that can be achieved through different processing technologies. Native polysaccharides are common candidates for nano carriers as they are bio-based, economically viable and biodegradable [29, 30]. However, they are hardly compatible with hydrophilic pesticides. Direct entrapment of pesticides with polysaccharides would result in low loading efficiencies. Kumar and co-workers [31] synthesized an imidacloprid loaded sodium alginate nanoparticles via an emulsion crosslinking technology. For that they prepared a secondary water–oil-water emulsion, in which pesticide and alginate was dissolved together in the inner water phase that was solidified via sodium-calcium ion exchanging to form nanoparticles. However, the loading efficiency was low (< 3%). Alternatively, the carriers can be chemically modified with hydrophobic groups for improved chemical compatibility. Li et al. [32] fabricated a series of avermectin-loaded microcapsules with starch acetate as the carrier matrix. Starch acetate can be co-dissolved with avermectin in dichloromethane (DCM) with composite nanoparticles formed using a premix membrane emulsification (PME) technology, and the loading efficiency was estimated in the range of 16 ~ 47%. Simultaneously employing hydrophobically modified polysaccharide and assisting surfactants has also been reported. Chun and Feng [33] reported the synthesis of abamectin-carrying nanoparticles by using hypromellose acetate succinate (HPMCAS) with lecithin as the amphiphilic stabilizer. The entrapment efficiency of abamectin was over 90% with active loading in the nanoparticles reached 50% by using nanoprecipitation (Fig. 2a). Polysaccharides can also help to stabilize the pesticides in addition to achieve sustained release. Chun and Feng demonstrated that the HPMCAS nanopesticides could maintain 75.6% of the abamectin activity after 40 h UV irradiation, attributing to the UV-barrier property of lecithin. As far as stability is concerned, the excellent UV-blocking property of lignin was believed effective in protecting pesticides against photodegradation [34, 35]. Li and co-workers [36] fabricated an avermectin-lignin nanopesticide with the anionic surfactant sodium lignosulfonate and cationic surfactant cetyltrimethylammonium bromide (CTAB) through electrostatic self-assembly method, where the entrapment of pesticide was completed via partial disintegration of the self-assembled structures in a pesticide-dissolved organic solvent and subsequent structural restoration in an aqueous phase (Fig. 2b). Avermectin loading efficiency in the final formulation was about 71%. In a more recent work, Zhou et al. [37] reported the fabrication of λ-cyhalothrin-entrapped nanopesticides with benzoyl lignin via a nanoprecipitation method with active agent loading reached of 64%.

Biodegradable synthetic polymers, specially polylactic acid (PLA) and poly(lactic-co-glycolic acid) (PLGA) are also attractive candidates for nano entrapment. They can be eventually degraded into environment-friendly lactic acid and glycolic acid monomers, while their hydrophobic nature also offers a good affinity to pesticide molecules [38, 39]. Employing PLA as the entrapping matrix, Liu and co-workers [40] prepared porous microspheres via a PME process to achieve sustained release of pesticide chlorantraniliprole. The entrapment efficiency of chlorantraniliprole reached 93.6%. In another work, a pyraclostrobin-loaded PLGA nanopesticide with a core–shell structure was synthesized by Zhong et al. [41] using a microfluidic technology. A mixture of poly(vinyl alcohol) (PVA) and pyraclostrobin suspension formed the inner core liquid, PLGA functioned as the outer shell, with entrapment efficiency measured as 93.9%. The author demonstrated that the size and shell thickness of the microcapsules could be controlled by varying processing parameters to achieve different release rates. The synthesis strategy and action mechanisms of above PLGA- and PLA-based nanpesticides are illustrated in Fig. 2c.

Compared to native polymeric materials, synthetic polymers can offer better processing flexibility in processing and structural designs [42–46]. Zhao et al. [42] fabricated a series of nanoscale pesticide-loaded “hat”-shaped Janus carriers (HJCs) via emulsion interfacial polymerization. Four pesticides of difenoconazole, prochloraz, pyraclostrobin, and tebuconazole were tested, and the HJCs showed distinct physical characteristics in the convex and concave areas on leaves driven by the “hanger-hat” topology and that consequently led to long-term retention and a stronger flush resistance. In another work, Zhang et al. [43] synthesized biodegradable castor oil-based polyurethanes using a prepolymer dispersion method, with diameters of nanoparticles could be manipulated to be below 50 nm and an entrapment efficiency > 85%.

Interestingly, some inorganic materials can also be applied to entrap pesticide molecules. Calcium carbonate nanoparticles carrying validamycin were prepared through a reversed-phase microemulsion process by Qian et al. [47]. Calcium carbonate was formed by the reaction between calcium chloride and sodium carbonate in an aqueous microemulsion stabilized by CTAB, with particle size in the range of 50 to 200 nm. The inorganic nanopesticide reached an entrapment efficiency of ~ 20% and remained active for 2 weeks after application.

#### Nanopesticides prepared by physical adsorption

Nanomaterials, especially those with porous structures, generally have large specific surface areas which can be translated into strong adsorption capacities to load active ingredients. Limited with processing flexibility, inorganic nanomaterials are more suited for processing with surface adsorption. For example, active carbon fabricated with polydopamine can efficiently adsorb water-soluble pesticide 2,4-Dichlorophenoxyacetic acid sodium with high active loadings [48]. Nuruzzaman et al. [49] reported the preparation of porous silica nanospheres with a large through hole for pesticides adsorption. It was believed that the large through hole endowed the system high loading capacities and desired release behaviors.

### Stimulated-release nanopesticides

**Fig. 3:**
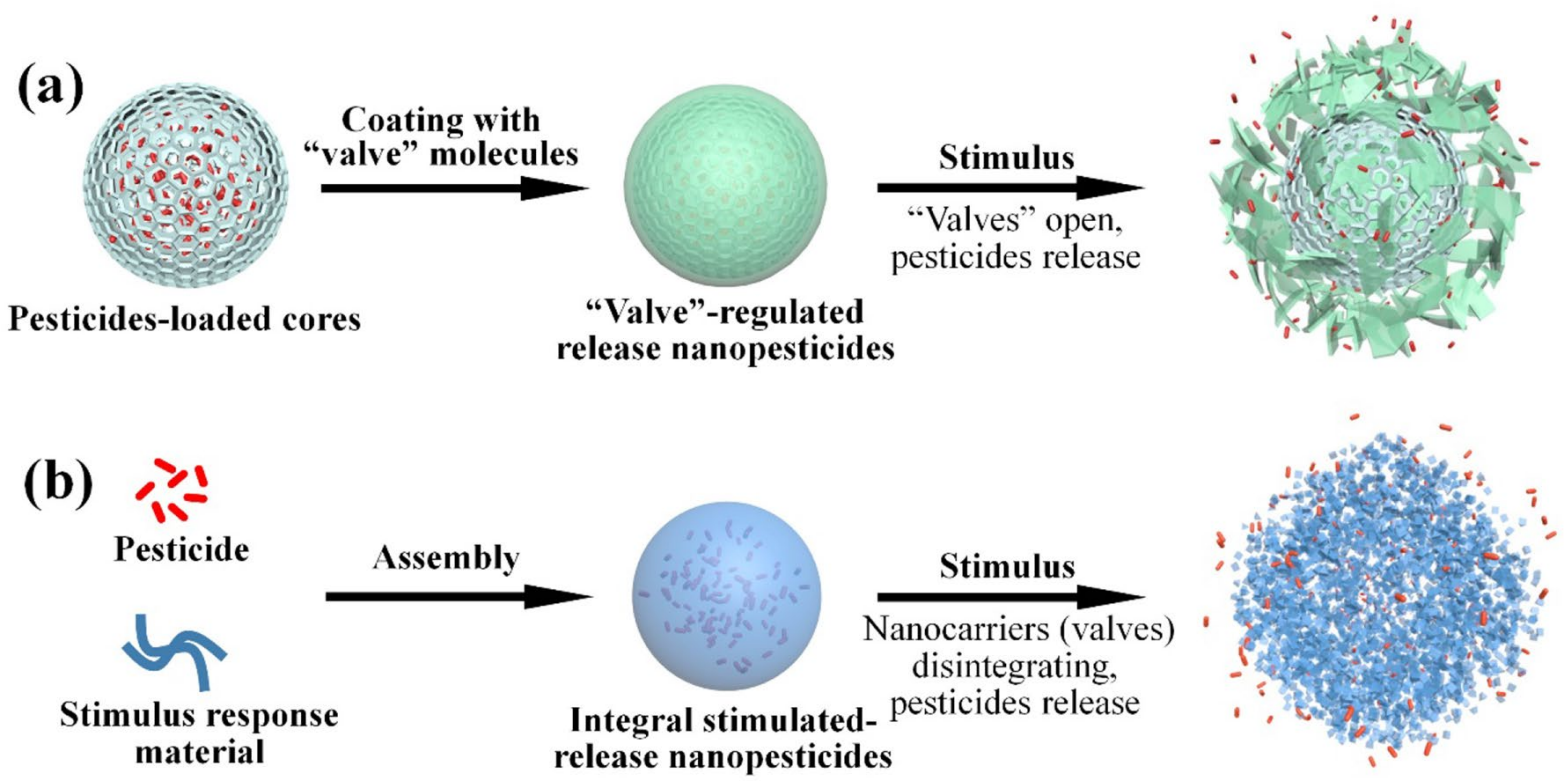
Typical Synthetic strategies and Action Mechanisms of Stimulated-Release Nanopesticides. **a** Valve-regulated release nanopesticides and **b** integral stimulated-release nanopesticides

Stimulated-release nanopesticides can realize site-specific and smart release of pesticides in response to biotic or abiotic stimuli [4]. The fabrication strategies of stimulated-release nanopesticides can be generally divided into two categories: (1) Valve-regulated release nanopesticides, which generally possess pesticide-loaded cores covered with a valve layer on its surface to block the release channel of the pesticides; upon exposed to specific stimuli that can induce biological, physical, or chemical reactions, valve blocking will be terminated and pesticide release gets initiated (Fig. 3a). (2) Integral stimulated-release nanopesticides, in which there is no obvious valve gating layer, the nanopesticide can respond to specific stimulus as a whole to initiate the release of pesticides (Fig. 3b). Table 2 summarizes typical preparation of stimulated-release nanopesticides reported recently.

**Table 2:** Typical fabrucation and preparation of stimulated-release nanopesticides

Category	Carrier Material/ active ingredient	Fabrication Method	Stimulation	Refs.
Valve-Regulated Preparation	Mesoporous Silica (core), PhAPTMS and α-Cyclodextrin (valve) /Chlorantraniliprole	Pesticide physically loaded in core structure; blocked by supramolecular structure formed by valve chemicals	α-amylase in insect intestine hydrolyzes α-cyclodextrin to open the valve	[50]
	HCMs (core), PEG and α-Cyclodextrin (valve) /Imidacloprid		Infrared light increases the system temperature and disrupt the valve for the photothermal effect of HCMs	[51]
	Attapulgite in Biochar (core), ASO and Azobenzene (valve)/ Glyphosate	Pesticide physically loaded in cores; blocked by ASO layer	UV–Vis light induces reversible cis–trans isomerization conversion of azobenzene, disturbing the ASO layer and promoting pesticide release	[52]
	NH_4_HCO_3_ containing Attapulgite (core), ASO and PVA (valve)/Glyphosate		Rising temperature, decomposes NH_4_HCO_3_ to produce CO_2_ and NH_3_ bubbles and generating micro/nano pores in the valve layer for pesticide release	[53]
	BNNP (core), PEG (valve)/Avermectin	Avermectin physically adsorbed in PEG-conjugated BNNP	PEG units are detached under strong alkaline condition to facilitate avermectin release	[54]
Integral Stimulated-Release	Graphene Oxide/ Cyhalothrin, Bifenthrin and fFenpropathrin	Physical Adsorption	Rising temperature facilitates pesticides release	[55]
	Chitosan/Spinosad	Chitosan microparticles formed via coprecipitation, spinosad loaded via physical adsorption and adhesion	Protonation of amino groups of chitosan in acidic condition causes a gradual solubilization of the chitosan microparticles to release spinosad	[56]
	Oligomeric Imine Based Surfactant/ Hydrophilic and Hydrophobic pesticides	The pesticides entrapped in worm-like micelles formed by surfactant molecular assembling	The imine groups of the surfactant could be hydrolyzed in acidic environment created by CO_2_ to release pesticides	[1]
	APTES and TEOS /Kasugamycin	Kasugamycin was conjugated with APTES and then forming pesticide-contained silica NPs via sol–gel method	Amidase in pathogenic microorganisms could disintegrate the nanopesticide to release kasugamycin	[57]
	pH-Jump Reagent 2,4-Dinitrobenzaldehyde and Zeolitic Imidazolate Framework-8 (MOF)/Prochloraz	In situ addition of prochloraz and pH-jump reagent in the synthesis process of the MOF structure	UV light makes pH-jump reagent to acidify the environment, interrupting the MOF structure to release prochloraz	[65]

#### Valve-regulated release nanopesticides

Valve-regulated release has been mostly realized by using inorganic core carriers with various designs of valving layers. In a recent work, mesoporous silica was applied as the carrier core for pesticide chlorantraniliprole [50], that was then covalently grafted to form a supramolecular structure with N-phenylaminopropyltrimethoxysilane (PhAPTMS) and α-cyclodextrin on the surface as the valve layer. The surface cover could be enzymatically hydrolyzed by α-amylase in insect intestine and causing chlorantraniliprole released to kill insects. With a similar design, Liu et al. [51] loaded imidacloprid to hollow carbon microspheres (HCMs) which were then capped with polyethylene glycol (PEG) and α-cyclodextrin. Because of the photothermal effect of HCMs, the valve layer could be disrupted when the system was exposed to near infrared light, initiating the release of imidacloprid. Chen et al. [52] developed another light-responsively valve-regulated release nanopesticide by using a two-stage physical adsorption method of glyphosate in attapulgite in biochar as the core, amino silicon oil (ASO) and azobenzene formed the surface valve layer. The reversible cis–trans isomerization conversion of azobenzene under UV–Vis light could disturb the ASO layer and thus promote the release of glyphosate. Chi et al. [53] mixed NH_4_HCO_3_ and glyphosate adsorbed attapulgite to form the carrier core, which was then physically coated with ASO and PVA as a valve layer to block glyphosate. When exposed to elevated temperature, NH_4_HCO_3_ would be decomposed to produce CO_2_ and NH_3_ bubbles, generating micro/nano pores in the valve layer to release glyphosate. Hao et al. [54] covalently modified avermectin adsorbed boron nitride nanoplatelets (BNNP) with valve molecule poly(ethylene glycol) diacrylate via esterification, PEG could prevent the release of avermectin under acidic and neutral conditions due to the steric effect; while under strong alkaline conditions, ester hydrolysis would detach the PEG units to facilitate avermectin release.

#### Integral stimulated-release nanopesticides

The stimulation-responding ingredients can also be integrated along with the active agents and carrier materials throughout the whole structure via simple processes such as physical adsorption. For example, Gao et al. [55] adsorbed three different pesticides to the surface of graphene oxide, which also function as the stimulation-responding component and could regulate pesticide release according to changes in temperature. More integral stimulated-release nanopesticides were reported in form of entrapment. Lin et al. [56] synthesized a spinosad-entrapped chitosan microparticles via a coprecipitation method, and the pesticide was entrapped in the carrier through physical adsorption and adhesion. When exposed to acidic conditions, the protonation of amino groups in chitosan would cause a gradual solubilization of the chitosan matrix, resulting release of spinosad with regulated rates for 6 days. Liu et al. [1] synthesized an oligomeric imine-based surfactant, which could be assembled into worm-like micelles and could entrap both hydrophilic and hydrophobic pesticides; once applied, the materials would absorb CO_2_ from air and create an acidic environment, hydrolyzing the imine groups and initiating the release of active loadings.

Covalent grafting was also employed for the fabrication of integral stimulated release nanopesticides. Ding et al. [57] silanized a carboxy-contained kasugamycin molecules with 3-aminopropyltriethoxysilane (APTES) via amidation and subsequently employed a sol–gel method to achieve a silica-based nanopesticide. The covalent structure could prevent the photodegradation of kasugamycin, but could be disintegrated by amidase produced by pathogenic microorganisms, leading to quick release of kasugamycin (~ 80% release was achieved with 14 h).

### Nanopesticides based on metal–organic framework (MOF) materials

Driven by the increasing environmental and health concerns, research in this area particularly seeks the development of nanomaterials from biodegradable and eco-friendly materials. It is noteworthy that there is a growing interest in using MOFs. MOFs are porous inorganic–organic hybrid material with typical frameworks consisting inorganic metal centers and organic ligands [58]. MOFs could afford broad ranges of physical and chemical properties in addition to extremely high specific surface areas, and key components can be selected from a wide range of eco-friendly materials. In fact, it is believed that most MOFs can be ultimately decomposed to components to be absorbed by soil as nutrients [59]. Physical adsorption of active ingredients to pre-made MOFs is the most common strategy for nanopesticides fabrication using MOFs. Studies have demonstrated that zirconium-, aluminum-, and iron-based MOFs synthesized via hydrothermal or microwave heating methods could achieved controlled release of various pesticides [59–61]. Additional regulation on release rate can be achieved by introducing additional components. In a work reported by Gao et al. [60], to prevent the premature escape of active ingredients from MOFs, a silica shell was introduced to cover pesticide-carrying MOFs. Similarly, Shan et al. coated polydopamine on diniconazole loaded MOF carrier so that the fungicide could be released in different rate according to pH changes in the environment [62]. In another work, Fe_3_O_4_-MOF core–shell nanocarriers was synthesized to physically adsorb imidacloprid [63]. Since they are magnetic, the nanoparticles were expected to be retrieved magnetically after active loadings are released, minimizing environmental impacts of the carriers and the residual pesticide. Entrapment of pesticides in situ of MOF synthesis could also be realized. Mejías et al. [64] synthesized bioherbicides-carrying zinc zeolitic imidazolate MOFs via an in situ hydrothermal method in which the bioherbicides were added in the reaction medium during the MOF formation, followed by surface modification with hydroxypropyl-β-cyclodextrin. The lifetime of the natural bioherbicides was prolonged with an eightfold enhancement in water solubility. The nanopesticide showed desired growth inhibition against weeds including *Lollium rigidum Gaudin*, *Echinochloa crus-galli (L.)* and *Amaranthus Viridis*. Applying a similar one-pot in situ synthesis method, Lang et al. [65] simultaneously entrapped fungicide prochloraz and a pH-jump reagent 2,4-dinitrobenzaldehyde in zeolitic imidazolate framework-8 to produce an integral stimulated-release nanopesticides. Under UV light irradiation, 2,4-dinitrobenzaldehyde acidified the environment and interrupted the MOF structure to release the prochloraz, showing an anti-fungal efficacy of ~ 51%, whereas the effectiveness of conventional prochloraz emulsion was only 9%.

## Nano-fabricated fertilizers

Different formats of nano-fabricated fertilizers were developed using a variety of natural and synthetic materials, with overall goals to achieved regulated release rate and high uptake efficiency to match crop growth needs. We may classify nano fabrication fertilizers into three categories: (1) Nano-Supported Fertilizers, in which nanostructured materials are applied as additives to regulate release of fertilizers, (2) Nanosized Fertilizers, refer to fertilizers made in nanoscale, and (3) Nano-Wrapped Fertilizers, which apply nanomaterial wraps or coatings to contain regular size fertilizers. Table 3 summarizes recently reported nano-fabricated fertilizers according to this classification.

**Table 3:** Typical nano-fabricated fertilizers

Category	Materials for nanostructure	Fabrication strategy/ Concrete method	Nutrient-release Mechanism	References
Nano-Supported Fertilizers	Calcium Phosphate	Entrapment/Doping nutrients into the nanocarrier formation system	Diffusion	[66–71]
	Chitosan and Anionic Compounds	Entrapment/Electrostatic self-assembly	Diffusion and Chitosan hydrolysis	[72–75]
	Liposome	Entrapment/Solvent-injection techniques or thin lipid-film hydration and extrusion methods	Integrity disruption caused by osmotic pressure	[76, 77]
	Nanofibers with PVA cores and PLA shells	Entrapment/Co-axial electrospinning	Diffusion & PLA shell hydrolysis and peeling	[78]
	Ethylene Oxide/Propylene Oxide Block Copolymer and Porous Palygorskite Nanoparticles	Entrapment/Fe nutrient physically adsorbed into palygorskite nanoparticles and then coated with the copolymer to block the nutrient	Temperature-stimulated release by utilizing the temperature-sensitive property of the copolymer	[80]
	Carboxyl Cellulose	Entrapment/Chelation of carboxyl cellulose and Fe^2+^	pH-stimulated release, the nanostructure would be disintegrated in acidic condition	[81]
	Porous Halloysite Nanotubes & Chitosan	Entrapment/Urea was physically adsorbed into porous halloysite nanotubes which were further coated with chitosan to block the nutrient	Glutathione produced by crops could broke down chitosan	[82]
	Biochar	Entrapment/physical adsorption	Diffusion	[83–85]
	Inorganic Porous Materials: zinc aluminosilicate, zinc layered hydroxide-nitrate, Zeolite, etc	Entrapment/physical adsorption	Diffusion	[86–88]
Nanosized Fertilizers	HA & Organic Acids	Neutralization of Ca^2+^ and PO_4_^3−^, organic acids could be functionalized by dipping	Dissolving promoted by nanometerization and organic acid-functionalization	[89–91]
	HA & Urea and Thermoplastic Starch	Mixing	Dissolving promoted by nanometerization and the soluble host matrixes of urea and starch	[92]
	Leonardite Potassium Humate & Fe_2_(SO_4_)_3_	Coprecipitation	Slow dissolving	[93]
	Manganese Zinc Ferrite Nanoparticle	Template-free microwave-assisted hydrothermal synthesis technique	Slow dissolving	[94]
	Metal–Organic Framework (MOF)	Hydrothermal method, microwave method, etc	Slow dissolving	[95–99]
Nano-Wrapped Fertilizers	Nano-Silica	Spraying the mixture of nano-silica and coating polymer on the surface of regular size urea	Reducing the porosity of coating through -OH cross-linking, thus extending release longevity of the coated urea tablet	[100]
	Nano-Silica and Nano Lauric Acid Copper	Spraying nanomaterials such as nano-silica and nano lauric acid copper on the surface of polyurethane coated urea tablets	Nanomaterials endow coating surface super-hydrophobicity, avoiding direct dissolution of urea by liquid water	[101–103, 108]
	Sodium Alginate-Loaded Hollow Nano-Silica	Sodium alginate-loaded hollow nano-silica was electrostatically adsorbed on the polyurethane coating of regular size urea	The sodium alginate would release to form gel with Ca^2+^, thus blocking the pores and cracks of the coating to regulate the release rate	[109]

### Nano-supported fertilizers

Fertilizers incorporated with nanostructure additives constitute probably the most extensively studied subject in the area of nano agrochemicals, with various preparation and functioning concepts reported recently. That may be classified into two major formats: (1) Entrapment Nanofertilizer, with nutrients dopped, encapsuled or entrapped in nanocarriers with hindered exposure, and (2) Adsorption Nanofertilizer, with nutrients incorporated into nanocarriers mainly through physical adsorption.

**Fig. 4:**
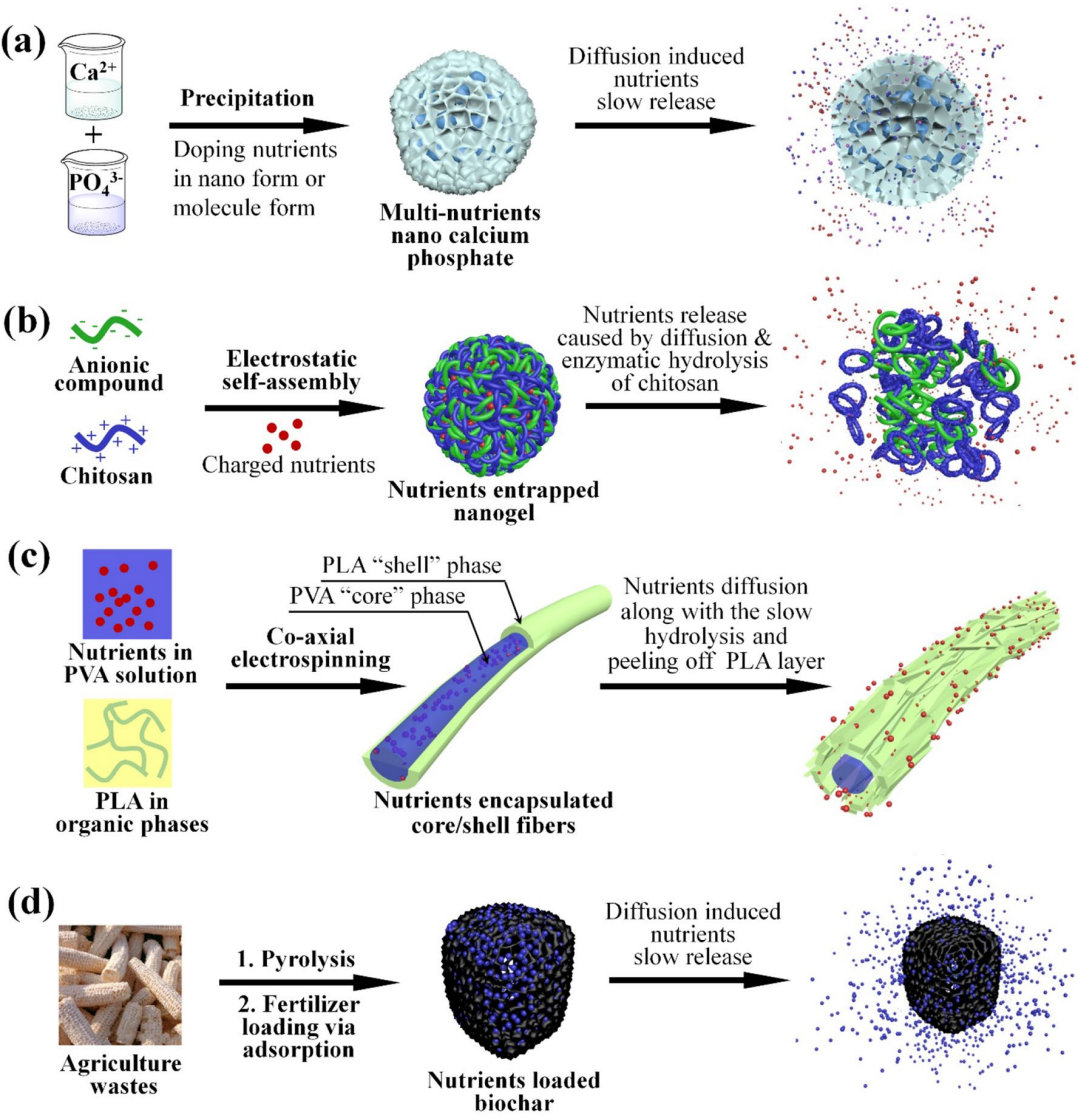
Fabrication Strategies and Release Mechanisms of Typical Nano-Supported Fertilizers. **a** Multi-nutrients nano calcium phosphate (HA and ACP) fabricated with doping method [66–71]; **b** Chitosan-cased nutrients entrapped nanogels fabricated via electrostatic self-assembly [72–75]; **c** Nutrients encapsulated core/shell nanofibers fabricated with co-axial electrospinning technology [78]; (d) Biochar-based nanofertilizer [83–85]

Entrapping fertilizer in nanoscale structures is a very common approach to prepare nanofertilizers. Kottegoda et al. [66] synthesized urea-hydroxyapatite (HA) nanohybrids with a urea to HA ratio of 6:1 by adding H_3_PO_4_ solution to a suspension containing Ca(OH)_2_ and urea. The amine groups of urea and the carbonyl groups of HA could form strong N–C-N bonds in the nanohycbrids, thus causing a slow release of urea up to 1 week in aqueous medium. In a more recent work, Tarafder et al. [67] synthesized a hybrid nanofertilizer by doping HA nanoparticles with urea, Cu(OH)_2_, Fe(OH)_2_, and Zn(OH)_2_, which could continuously release nutrients for more than 14 days, and the required fertilizer dosages could be reduced to about 1% of conventional fertilizers. Besides HA nanoparticles, amorphous calcium phosphate nanoparticle (ACP) was recently employed for controlled release of urea [68–71] by doping urea onto ACP (Fig. 4a). The large specific surface areas of ACP allowed simultaneous release of Ca and P to crops at desired release rates.

Bio-based materials such as chitosan are also promising nano additives. The amino residue groups of chitosan offer positive charges in acidic environments, so that it can be nanosized through ionic gelation with anionic compounds. The release rate of nutrients entrapped inside chitosan nanogels could be controlled either by mass transfer resistance manipulations or by enzymatic hydrolysis of chitosan by enzymes from targeted plants [72–75] (Fig. 4b). Liposome nanoparticles that have been used widely in biomedical engineering have also been employed as fertilizers carriers [76, 77]. Nutrients could be loaded into liposome system by using well established solvent-injection techniques or thin lipid-film hydration and extrusion methods. Once reached crop leaf stomata or root systems, the integrity disruption caused by osmotic pressure could lead to intracellular release of nutrients. Nanofibers have also been employed to fabricate entrapment nanofertilizers. Nooeaid et al. [78] loaded the conventional NPK fertilizer into core/shell nanofibers via co-axial electrospinning, where PVA was applied in the core phase along with active ingredients loadings, while hydrophobic PLA formed the shell phase (Fig. 4c).

Similar to designs developed for nanopesticides, stimulated-release fertilizers could better regulate nutrients release behaviors than passive release, thus offering improved matching with uptake characteristics of crops [79]. Considering that the demand of crops for Fe follows a low–high-low pattern as the ambient temperature rises, Chi et al. [80] employed ethylene oxide/propylene oxide block copolymer to entrap Fe in porous palygorskite nanoparticles. The copolymer demonstrated a temperature-sensitive nature by forming a liquid state at a temperature window of 25–35 °C and gel a state at 15 ~ 45 °C. The release rate of Fe could be adjusted to match that of crop absorption. In another work, Fe^2+^ was carried by carboxyl cellulose via a chelation process, produced nanomaterials that were sensitive to pH changes and could be broken down in acidic conditions [81]. Wang et al. [82] loaded urea in the porous halloysite nanotubes which were further coated with chitosan. Once applied, the disulfide bonds of chitosan could be broken down by glutathione produced by crops, therefore significantly enhancing the release of urea inside crops.

Adsorption nanofertilizers are preferred for formulations using porous or laminated inorganic or carbonaceous nanomaterials. In particular, biochar nanocarriers produced by carbonization of agriculture wastes or low-value biomass has drawn lots of attention, attributed mostly to their low cost and outstanding physical adsorption capacities [83] (Fig. 4d). Controllable release kinetics of a variety of nutrition ingredients including N, P, K, Na, Mg, Ca and Zn have been demonstrated in biochar-based adsorption nanofertilizers. One additional attractiveness of biochars is their ability to act as a soil conditioner due to their excellent swelling capacity, offering a water retention ability benefiting the soil [83–85]. Inorganic materials including mesoporous zinc aluminosilicate (ZnAl_2_Si_10_O_24_) [86] zinc layered hydroxide-nitrate and zinc layered hydroxide phosphate [87] have also been employed as adsorption supports for N, Zn and P delivery. Zeolite offers appealing high porosity, and has also been applied to construct nanocomposites with Fe_2_O_3_ for Fe delivery [88].

### Nanosized fertilizers

Insoluble nutrients such as minerals can be made in nanoscale to increase their adsorption by crops. HA nanoparticle has been demonstrated to be able to increase P uptake efficiency [89]. Based on that, more powerful and enriched fertilizers were made by further modification with organic acids [90, 91] or dispersing in matrix of urea and thermoplastic starch [92]. In another work, iron-humic nanosized fertilizer synthesized by Ceischi et al. [93] showed an enhanced Fe uptake, and thus reducing the Fe deficiency symptoms of soybean plants in iron-deficient calcareous soil. Due to the small size of the nanosized fertilizers (smaller than the sizes of leaf stoma), they are particularly favored for foliar fertilizers, which can be directly uptaken by plants and avoid the drawbacks of soil application [76]. For instance, Shebl et al. [94] reported the fabrication of manganese zinc ferrite nanoparticles as foliar fertilizer via a template-free microwave-assisted hydrothermal synthesis technique. When applied for growth of squash (*Cucurbita pepo L*), and the highest yield of squash plant increased by 52.9% in comparison to untreated squash. Recently, several hybrid MOFs, including iron-based MOF, oxalate-phosphate-amine MOF, and urea/iron MOF, have been synthesized as nanosized fertilizers [95–99].

### Nano-wrapped fertilizer

**Fig. 5:**
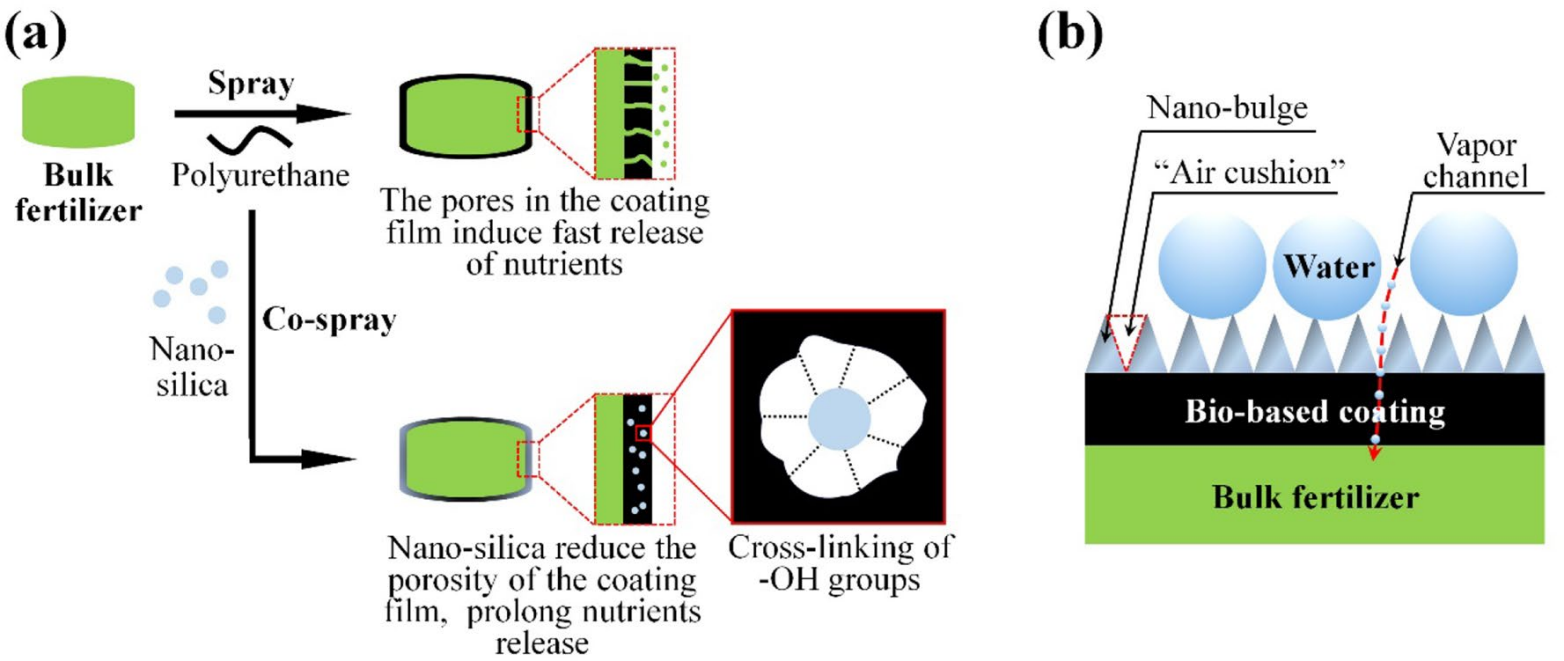
Representative Mechanisms for Preparation of Nano-Wrapped Fertilizers. **a** Nano-silica induced cross-linking reducing the coating porosity [100]; **b** Dense nano bulges inducing surface superhydrophobicity [101–103]

The main goal of wrapping conventional regular size fertilizers is to protect against water dissolution and thus reduce nutrient loss. Traditional petroleum-based wrapping materials are usually hydrophobic, while majority of bio-degradable materials are hydrophilic, both presenting difficulty for release control [79, 100–107]. Formation of composite wraps or coatings is therefore desired. In one of the works published by Yang et al., nano-silica was added to coatings made of bio-polyol and methylene diphenyl diisocyanate (MDI) to reduce the porosity through -OH cross-linking, thus extending release longevity of the coated urea tablet [100] (Fig. 5a). They further refined the coating preparation by spraying nanomaterials such as nano-silica [100, 108] and nano lauric acid copper [101], alone or together with hydrophobic molecules [102] on the surface of polyurethane coated urea tablets. The nanomaterials could block the micro-holes of the polymer coatings in addition to endow surface superhydrophobicity, avoiding direct dissolution of urea by liquid water. The porous structure would allow penetration of water vapor, thus achieving sustained and coating structure-regulated release (Fig. 5b).

In addition, Yang’s group also proposed a “film damage repair” strategy to sustain the release of fertilizers [109]. For that, bio-based polyurethane coatings were modified by polyethynimine and dopamine hydrochloride through a layer-by-layer method, and followed by further modification with sodium alginate-loaded hollow nano-silica. The sodium alginate would subsequently release to form gel via crosslinking mediated by calcium ions, thus blocking the pores and cracks of the coating to regulate the release rate.

## Nano activity-based growth promoters

Nanoparticles afford unique activities associated with their size traits that provide a variety of mechanisms to promote growth and health of agronomic plants. Such size-dependent nanoscale activities are mostly enabled by their high surface energy and high diffusivity in specific microenvironments, and in some cases via chemical reactivities (mostly indirect and non-corrosive activities against plants or environment, such as ionization of Ag NPs). The nanoscale size allows the materials function in micro-scale environments, garnering material-plant stimulation effects. Such materials function either through alternation of crop physiology, or by alleviating environmental stresses [110, 111]. Specifically, that may include (1) improving plant tolerance against environmental stresses, (2) altering or improving the function of plant tissue or organelle, or (3) remediating toxic pollutants.

Several types of active nanoparticles have been explored recently, focusing mostly on metal, metal oxide and bio-chemical nanoparticles. Various silver nanoparticles are probably the most active and extensively studied, followed by oxide particles including zinc, magnesium, cerium, calcium, and iron. The use of bio-chemical nanoparticles is probably the most recent development in this area. Very interestingly, magnetic nanoparticles have also fund strong effects on seed germination.

### Metal nanoparticles

The most extensively examined pure metallic nanoparticles are Ag NPs. In addition to its antimicrobial activity, Ag can also impact physiology of plants and function as a growth promoter. A recent study showed Ag NPs (applied in levels up to 100 ppm) could help to increase oil content for thymus crops [112]. Also, Ag NPs synthesized via mediating of an endophytic fungus isolated from marine seaweed could function as a biomimetic growth promoter with doses as low as 5 ppm [113]. Many other studies examined effects on seedling development and seed germination of Ag NPs. In the study of Ag NPs on salt tolerance of Satureja hortensis L. during in vitro and in vivo germination tests, while control tests showed that a significant reduction in germination percent and seedling growth due to the salinity stress, the application of Ag NPs (up to 80 ppm) significantly improved samples’ salinity tolerance [114].

**Fig. 6:**
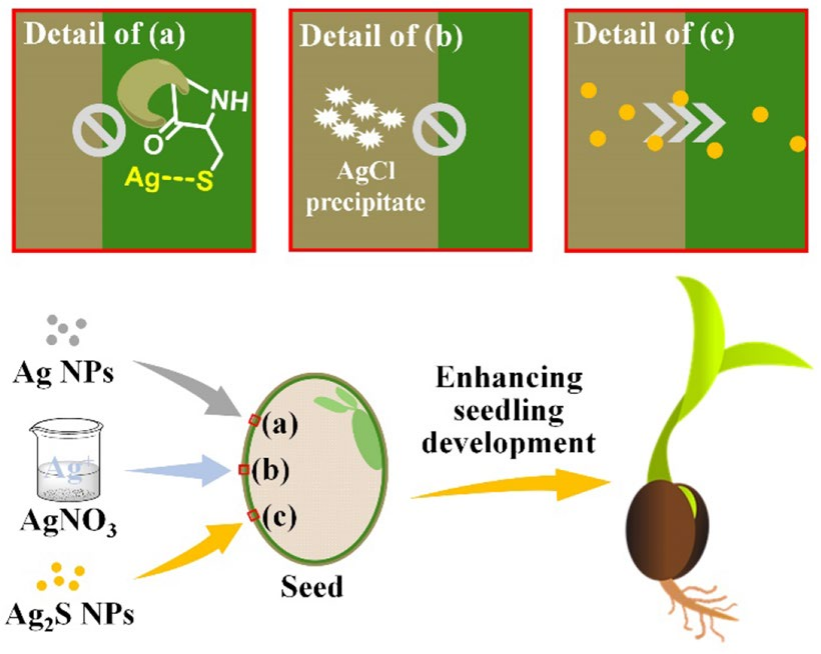
Potential Migration and Chemical Transformation of Ag NPs, AgNO_3_, and Ag_2_S NPs Across Seed Coats [115]. **a** Ag NPs forms thiolate complexes in the internal layer of seed coat (parenchyma cells); **b** AgNO_3_ would form AgCl precipitate in the external layer of seed coat (epidermis); **c** Ag_2_S NPs could cross seed coat without significant chemical modifications

Mechanisms of Ag NP activity is under investigation yet still in a very early stage of understanding. In a study of Ag NPs prepared in different forms, including Ag_2_S and AgNO_3_ in addition to Ag (up to 1000 ppm) on the germination of Phaseolus vulgaris seeds, Ag NPs interestingly did not affect the germination rate, but the development of seedlings was significantly improved by Ag_2_S NPs, while AgNO_3_ showed a negative effect compared to the control (water) [115]. The difference was attributed to chemical stability of the nanomaterials applied. While Ag NPs and AgNO_3_ were found transformed to chelate or AgCl precipitate in the parenchyma cells or epidermis of seed coat, thus could not get inside the seed (Fig. 6a and 6b), Ag_2_S NPs did not show any detectable chemical changes in the crossing process (Fig. 6c) [115]. The Ag activity could be closely associated with its antimicrobial activity. In a work examining silver-incorporated titanium dioxide nanoparticles (Ag-TiO_2_ NPs, 7 and 26 nm) for spinach seed treatment and spinach plant growth, it showed that the plant growth could be affected by particle concentration and size. The positive effects of Ag-TiO_2_ NP treatment were attributed to the generation of reactive oxidized species that can induce antimicrobial activities to retain a healthy microenvironment for plant growth [116].

Other metallic nanoparticles including Cu and Au NPs function in very similar ways as Ag NPs. Cu NPs were examined recently for plant growth promotion. In a study on the antifungal activity of copper nanoparticles (Cu NPs) against the beetle/fungus complex and their effect on the beetle’s reproduction, it was revealed that pure metallic Cu with an average size of 240 nm could be potentially considered as an alternative for the control of the beetle-fungi complex or even be integrated into novel disease management strategies [117]. Au NPs have also been used as a deliver vehicle for other promoters or stimulators. It was reported that Au NPs (20–22 nm) charged with harpin successfully induced defense responses in tobacco [118]. Ca NPs were also prepared and examined, found effective on crocin, picrocrocin, and safranal content [119]. Especially in combination with putrescine, Ca NPs could increase growth and phytochemical properties in Crocus sativus [119].

### Metal oxide nanoparticles

Zinc oxide nanoparticles (ZnO NPs) were examined and showed promising functionalities. Recent studies have revealed the uptake, distribution and the effects of ZnO NPs in plant physiology [120]. Foliar exposure of ZnO NPs improved the growth of wheat (Triticum aestivum L.) and decreased cadmium concentration in grains under simultaneous Cd and water deficient stress [121]. In a different study, biogenic zinc oxide nanoparticles (ZnO NPs) using an extract of a wild and spontaneous aquatic species, Lemna minor (duckweed), showed evident effectiveness for growth stimulation, with stimulated contents of chlorophylls, carotenoids, and anthocyanin [122]. Both ZnO and TiO_2_ NPs can function as insecticidal agents, due to their oxidation activities [123]. In a study with second-stage nymphs under laboratory and greenhouse conditions in tomato, direct spray of ZnO NPs, TiO_2_ NPs, and their combinations, showed promising potential for B. cockerelli control [123]. In another study, ZnO and Ag NPs showed potent antifungal activities against A. solani. [124]. ZnO NP can also generate Zn^2+^ ions, impact adversely on plant germ cells, such as pollen grains. The germination and tube elongation of pollen grain (Lilium longiflorum) exposed to low-solubility NPs was observed [125], attributed to cells absorption of Zn^2+^ generated by the particles. The germination rate of pollen grain exposed to 100 mg/L ZnO NP dispersion decreased significantly from controls [125]. Despite the low solubility of zinc oxide nanoparticle, pollen cell-attached particles inhibited germination and elongation of pollen tube by continuous Zn^2+^ dissolution from particles and Zn^2+^ absorption by the cell [125].

Iron oxide magnetite nanoparticles showed interesting functionality as a plant growth stimulator. Magnetite nanoparticles coated with citric acid demonstrated apparent stimulation of soybean and alfalfa growth [126]. In that work, the internalization and distribution of citric acid-coated magnetite nanoparticles (here, Fe_3_O_4_ NPs) in the plants and their effects on plant growth were studied. These findings suggested that Fe_3_O_4_ NPs are readily absorbed but not translocated (soybean) or scarcely translocated (alfalfa) from the roots to the shoots, suggesting that the NPs behave as plant growth stimulators. In another study aimed to examine the morpho-ultrastructural impact of iron oxide (Fe_3_O_4_) NPs on seed germination in tobacco (Nicotiana tabacum var. Turkish), most NPs-treated seeds exhibited significant higher seed germination (except for seeds treated with NPs with sizes below 10 nm NPs). Thick and thin micrographs of radicles and leaflets of 5 nm NPs-treated seeds (30 mg/L concentration) and 10 nm NPs (30 mg/L concentration) showed structural and ultrastructural deformation. Thus, it was suggested that the toxicity and the bioaccumulation of Fe_3_O_4_ NPs were size and concentration dependent [127]. Magnetic NPs also showed remediation potentials in Cu-polluted soil–plant systems. Several positive environmental aspects relative to magnetic NP use, including the harmless effects of magnetic NPs on sunflowers (1% in soil) and the ability of magnetic particles to influence Cu mobility in the soil were reported (as reviewed in [128]). Decreased lipid peroxidation indicated an enhanced antioxidant enzymatic response of magnetic NP-exposed plants [128].

Other oxide NPs were also brought to attention. In a work to evaluate the use of nano-CeO_2_ on the biological and nutritional characteristics of Spodoptera frugiperda (an arthropod pest widely distributed in agricultural regions), results confirmed toxicity of nano-CeO_2_ for S. frugiperda under field conditions [129]. Additionally, nano-silica was also found effectively replacing chemical insecticides to protect stored products [130].

### Other types of NP promoters

There is a growing interest in developing eco-friendly, biodegradable, cost-efficient, and biopolymer-based nanohybrid constructs for plant growth promotion. In a recent report, foliar application of Chitosan nanoparticles (ChNPs) significantly enhanced the growth, yield, and mineral content (Fe, Zn, Mn, P, Ca, Mg) when compared to controls [131]. ChNPs also induced several defenses related enzymes (chitinase, beta-1,3 glucanase, chitosanase, protease inhibitors, peroxidase, polyphenol oxidase) in leaves of finger millet plants [131]. Application of salicylic acid nanoparticles (SA NPs) could induce some resistant genes of sweet pepper against black mold disease [132]. In vitro studies revealed that SA NPs applied at 1.4 mM significantly suppressed the growth of *A. alternata* [132]. Graphene also showed effects on the morphological and physiological regulative mechanisms in alfalfa, demonstrated growth promotion under abiotic stress [133]. The coupling effects of graphene and pH on plant growth, photosynthetic parameters and enzymes of the antioxidant defense system on leaves and roots were observed, and significantly promoted plant growth was detected [133].

Many other different types nanomaterials were examined to function as cleanup agents to remove hazardous environmental pollutants (as reviewed in [134]). Materials such as silica, non-magnetic/magnetic, carbon nanotubes/nanorods, nanoclay/nanomembrane, MOFs, graphene oxide, and other nanomaterials have been examined in combination with carrageenan biopolymers focusing on environmental remediation [134].

### Toxicity and environmental safety concerns

Potential toxicity of NP materials, especially bioactive NPs, to both environment and health are of full awareness and are being scrutinized closely. Toxic pollution when NPs are used as an assisted phytoremediation alternative has been reviewed recently [135]. Attention also paid on forms and types of nanoparticles and the pathways of their transmission in plants and those who take treated plants as foods [136]. Carcinogenicity of many NPs, especially metal nanomaterials, has been also reviewed for the researchers and policymakers in manufacturing industries and biomedicine [137].

So far, there are more than 100 pesticides that contain Ag due to its anti-microbial properties [138]. Properties associated with nanosized materials may also pose a threat to the environment since with the fate and lifecycle of nanomaterials remain poorly understood and largely uncontrollable. Possible consequences include phytotoxicity and genotoxicity due to the NPs and their transformation intermediate chemicals [139, 140]. Lu et al. have reported that the citrate-coated colloidal Ag nanoparticles were not genotoxic- (genetic), cytotoxic- (cell), and photo- toxic (toxicity through photo-degradation) to human; however, citrate-coated Ag nanoparticles in powder forms were toxic [141]. Nevertheless, a recent review analyzed the quantitative data on the input and content of silver nanoparticles (Ag NPs) and their possible transformations in soil [142]. It revealed that currently available data on the Ag NPs content in soil are exclusively based on simulation results and varied in a wide range from 5.33 × 10^–6^ to 7.4 mg/kg at an annual input rate of 1.2 × 10^–3^ to 9.68 mg/kg. Analysis of the existing concepts of the Ag NPs translocation from soil to plants suggested no current risk of contamination of agricultural products with Ag NPs. Some data demonstrated that negative effects of Ag NPs on microorganisms were also time-dependent, and it was suggested to assess the effects of Ag NPs in soil in long-term experiments (over 90 days) at the nanoparticle concentration not exceeding 10 mg/kg [142].

Similar to Ag NPs, the chemical activity of ZnO could also cause various detrimental effects in plants at high dose, which might vary with different plants as well as with the size and shape of ZnO NPs [120]. Extensive research has been conducted to overcome the antagonist effect of ZnO NPs, where low dose and duration of exposure are found to be beneficial. Nevertheless, it was believed that ensuring the stability of NPs can reduce the harmful impacts of ZnO NPs in plant and simultaneously enhance their promoting efficacy [120]. In addition, safety of TiO_2_ NPs was also examined [143–145]. The study of Larue et al. [145] showed that TiO_2_ NPs would accumulate in the plantlets of wheat and rapeseed. However, there is no systematic study yet to track the metabolism pathway of TiO_2_ NPs in crops.

## Summary and perspectives

The large number of publications on agricultural nanotechnology over the last several years clearly indicates tremendous endeavors are undertaking in this area. That has included contributions from scientist across a variety of disciplines such as material chemistry, biology, environment, health, information tech, in addition to agronomy and agricultural engineering. Nevertheless, it is a burgeoning area in an infancy stage, large-scale commercial applications of nano agrochemicals are yet reported. Among the potential challenges limiting the scalability of nano agrochemical technologies, several factors as to be discussed in the following are particularly outstanding.

The harsh and complicated, mostly outdoor environment of agricultural production may present potent challenges against chemical and structural stability of nano devices. Taking nanopesticides for examples, release kinetics studies examined under lab-manipulated operational conditions can be very different from in-field conditions going through seasonal weather stresses in terms humidity, temperature, wind, and UV exposure. Nanopesticides with magnetic Fe_3_O_4_ cores, which are considered retrievable to reduce the hazardous effects of the carriers and the residual pesticide [63], could lose the magnetic Fe_3_O_4_ cores due to structural interruptions under corrosive oxidation and photo attacks. Similarly, the valve opening threshold of the stimulated-release nanopesticides may also lose their sensitivity under in-field conditions.

The environmental and human health risks associated with nano agrochemicals have to be addressed thoroughly before any large-scale applications could be eventually considered. In addition to concerns as described above in the part of the toxicity and environmental safety of using nano agrochemicals, safety and environmental impacts during manufacturing processes will also come into play. The use of bio-based and inorganic materials that could be absorbed by soil without permanent negative impacts should be encouraged [146], and that has been the primary motivation of many research work in the area. In addition to that, how to handle large quantities of nano materials that are prone to generate nano dust emissions has to be considered eventually but has been largely ignored at this time to our opinion.

Finally, the economic viability of nano agrochemicals can also be challenging. That depends on both material selection and production cost. Compared to conventional agrochemicals, the nanomaterials require low application frequency and smaller doses by promising highly efficient performance, that should offer a good niche toward more affordable nano agrochemicals [147–149].

Overall, modern precision agriculture is a particularly cross-disciplinary area, and nanotechnology-based agrochemicals may eventually have to be associated with other smart technologies to meet the high demands and realize desired efficiency. By the end of the day, nano agrochemicals can only be successful by satisfying specific situation-sensitive requirements, including the nature of soil, the types and growth status of plants, climatic conditions, varying nutritional demands, etc.

## Data Availability

Not applicable.

## Abbreviations

DCM: Dichloromethane
PME: Premix membrane emulsification
HPMCAS: Hypromellose acetate succinate
CTAB: Cetyltrimethylammonium bromide
PLA: Polylactic acid
PLGA: Poly(lactic-co-glycolic acid)
PVA: Poly(vinyl alcohol)
HJCs: “Hat”-shaped Janus carriers
MOF: Metal-organic framework
PhAPTMS: N-phenylaminopropyltrimethoxysilane
HCMs: Hollow carbon microspheres
PEG: Polyethylene glycol
ASO: Amino silicon oil
BNNP: Boron nitride nanoplatelets
APTES: 3-Aminopropyltriethoxysilane
HA: Hydroxyapatite
ACP: Amorphous calcium phosphate nanoparticle
MDI: Methylene diphenyl diisocyanate
